# Structure–Function Relationships of Glycine and GABA_A_ Receptors and Their Interplay With the Scaffolding Protein Gephyrin

**DOI:** 10.3389/fnmol.2018.00317

**Published:** 2018-09-12

**Authors:** Vikram B. Kasaragod, Hermann Schindelin

**Affiliations:** Institute of Structural Biology, Rudolf Virchow Center for Experimental Biomedicine, University of Würzburg, Würzburg, Germany

**Keywords:** glycine receptors, GABA_A_ receptors, gephyrin, moonlighting protein, inhibitory post-synaptic specialization, cytoskeletal proteins

## Abstract

Glycine and γ-aminobutyric acid (GABA) are the major determinants of inhibition in the central nervous system (CNS). These neurotransmitters target glycine and GABA_A_ receptors, respectively, which both belong to the Cys-loop superfamily of pentameric ligand-gated ion channels (pLGICs). Interactions of the neurotransmitters with the cognate receptors result in receptor opening and a subsequent influx of chloride ions, which, in turn, leads to hyperpolarization of the membrane potential, thus counteracting excitatory stimuli. The majority of glycine receptors and a significant fraction of GABA_A_ receptors (GABA_A_Rs) are recruited and anchored to the post-synaptic membrane by the central scaffolding protein gephyrin. This ∼93 kDa moonlighting protein is structurally organized into an N-terminal G-domain (GephG) connected to a C-terminal E-domain (GephE) via a long unstructured linker. Both inhibitory neurotransmitter receptors interact via a short peptide motif located in the large cytoplasmic loop located in between transmembrane helices 3 and 4 (TM3-TM4) of the receptors with a universal receptor-binding epitope residing in GephE. Gephyrin engages in nearly identical interactions with the receptors at the N-terminal end of the peptide motif, and receptor-specific interaction toward the C-terminal region of the peptide. In addition to its receptor-anchoring function, gephyrin also interacts with a rather large collection of macromolecules including different cytoskeletal elements, thus acting as central scaffold at inhibitory post-synaptic specializations. Dysfunctions in receptor-mediated or gephyrin-mediated neurotransmission have been identified in various severe neurodevelopmental disorders. Although biochemical, cellular and electrophysiological studies have helped to understand the physiological and pharmacological roles of the receptors, recent high resolution structures of the receptors have strengthened our understanding of the receptors and their gating mechanisms. Besides that, multiple crystal structures of GephE in complex with receptor-derived peptides have shed light into receptor clustering by gephyrin at inhibitory post-synapses. This review will highlight recent biochemical and structural insights into gephyrin and the GlyRs as well as GABA_A_ receptors, which provide a deeper understanding of the molecular machinery mediating inhibitory neurotransmission.

## Introduction

**FIGURE 1:**
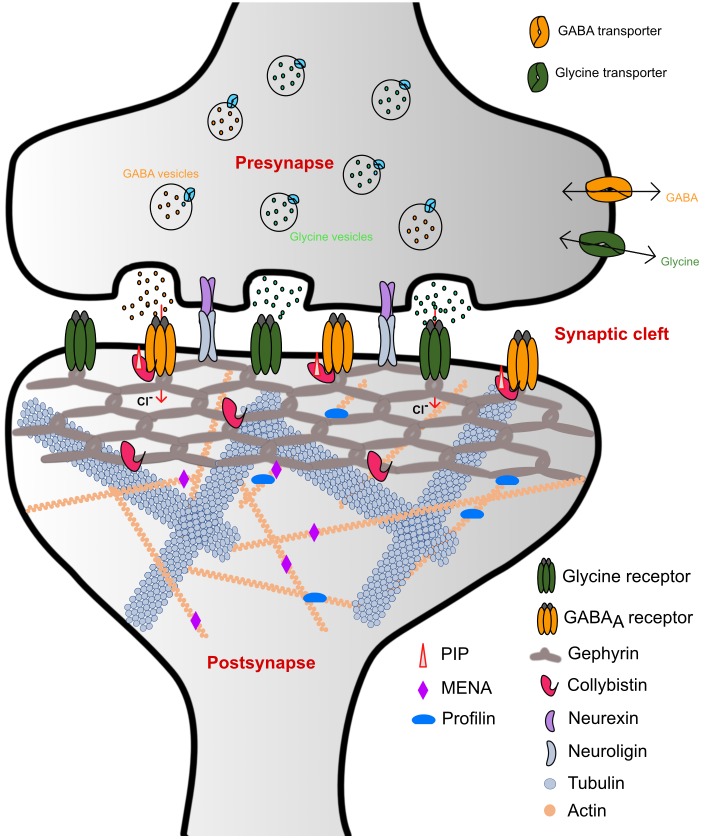
Schematic representation of inhibitory synaptic specializations. While the simultaneous presence of both GlyRs and GABA_A_Rs was chosen for illustrative purposes, such a mixed receptor population exists in spinal cord neurons (Dumoulin et al., 2000). Please note that many interaction partners of gephyrin including DLC and Pin1 have been omitted from the schematic to improve clarity.

Homeostasis of the brain is maintained through a complex interplay between excitatory and inhibitory neurotransmission. Synapses can be divided into two classes: chemical and electrical synapses, depending on the type of signal controlling neurotransmission. The chemical synapses, where a broad range of endogenous chemicals modulate neurotransmission, in turn, can be further subdivided into two general classes: (i) Excitatory synapses, where signal transmission is mainly mediated by cation-permeable receptors such as ionotropic glutamate receptors (iGluRs), and (ii) inhibitory synapses where neurotransmitter receptors such as the glycine and GABA_A_ receptors (GlyRs and GABA_A_Rs) reside, which are permeable to chloride (Cl^-^) ions (**Figure 1**).

GlyRs and GABA_A_Rs are the principal determinants of the majority of fast synaptic inhibitory processes in the central nervous system (CNS) (Betz et al., 1991). GlyRs containing the β-subunit and a subset of GABA_A_Rs are recruited and anchored at inhibitory post-synapses by the ∼93 kDa principal scaffolding protein gephyrin (Kirsch et al., 1991; Prior et al., 1992) (**Figure 1**). Initially, gephyrin was co-purified with microtubules during the isolation of GlyRs (Kirsch et al., 1991). Subsequently, biochemical, biophysical, and structural studies have shed light on the role of gephyrin in the process of recruitment and clustering of not only GlyRs but have also documented an obligatory dependence of a subset of GABA_A_Rs on gephyrin (Gunther et al., 1995; Sassoe-Pognetto et al., 1995; Craig et al., 1996; Kneussel et al., 1999a,b).

The inhibitory GlyRs and GABA_A_Rs neurotransmitter receptors can assemble into either hetero or homopentamers. GlyRs consist of five different subunit classes encompassing four α and a single β subunit. Heteropentameric receptors have been proposed to be composed of either two α and three β (Grudzinska et al., 2005) or two β and three α subunits (Langosch et al., 1988; Patrizio et al., 2017). These Cys-loop family members share a common structural architecture, which contains an extracellular domain featuring a twisted β-sheet composed of ten β-strands followed by four transmembrane helices and two intracellular loops as well as one extracellular loop connecting these helices. In contrast to the GlyRs, GABA_A_Rs are more diverse, consisting of 19 different subunit classes derived from eight different subunit types (α, β, γ, δ ε, π, ρ, and Θ). The most common GABA_A_Rs are composed of two α, two β, and single γ or δ subunit (Sigel and Steinmann, 2012). Amongst the heterogeneous pool of subunit classes of GABA_A_Rs, α1-3, β2-3, and γ2 subunits are localized at post-synaptic densities predominantly mediating phasic inhibition, where the inhibitory effects are transient in response to the high concentration of GABA as a consequence of the vesicular release of the neurotransmitter from presynaptic terminals. In contrast, the α4-6 and δ subunits are present at extrasynaptic sites where the concentration of the GABA is ambient, thus contributing toward tonic inhibition in the CNS. As dysfunctional inhibitory neurotransmission has been directly implicated in several neurological disorders, inhibitory neurotransmitter receptors have been a prime target of drug discovery efforts (Rudolph and Knoflach, 2011). A broad range of drugs including diazepams and also multiple sedatives as well as analgesics are currently in clinical use. Although biochemical and electrophysiological studies have helped us to understand multiple aspects of the regulation of these receptors, the complexity of the receptor composition in the pentameric assembly as well as the existence of multiple post-translational modifications hindered structural elucidation of the GABA_A_Rs and GlyRs. Hence, a clearer understanding of the structure of these ionotropic receptors (Miller and Aricescu, 2014; Du et al., 2015; Huang et al., 2017a) came into existence only quite recently.

The scaffolding protein gephyrin was shown to interact with post-synaptically localized GABA_A_Rs containing the α1-3 subunits (Maric et al., 2011; Mukherjee et al., 2011; Tretter et al., 2011), α5 subunit (Brady and Jacob, 2015) and also possibly those containing the β2-3 subunits (Kowalczyk et al., 2013). In contrast, although the GABA_A_R γ2 subunit does not bind to gephyrin directly, it has been shown to be essential for the clustering of GABA_A_Rs and gephyrin at the post-synaptic membrane (Essrich et al., 1998). In contrast to the GABA_A_Rs, the β subunit of the GlyR is the only GlyR subunit that interacts with gephyrin. In addition to the clustering of the receptors, gephyrin also associates with several other macromolecules including: (i) Collybistin, a guanine nucleotide exchange factor; (ii) Neuroligin 2, a cell adhesion molecule; (iii) Actin-associated proteins such as Profilin1 and 2 as well as IQSEC3. Together with these interactors gephyrin functions as the principal organizer at inhibitory post-synaptic specializations (also reviewed in Tyagarajan and Fritschy, 2014; Choii and Ko, 2015). Structurally, gephyrin is composed of ordered N and C terminal domains, referred to as GephG and GephE, respectively, which are connected by a flexible linker.

Gephyrin is a prime example of a moonlighting protein (Feng et al., 1998; Copley, 2003). Moonlighting proteins are multifunctional proteins, which carry out two or more functions, which are often independent of each other (Copley, 2003; Huberts and van der Klei, 2010). In the case of gephyrin, in addition to its receptor-anchoring function, in its phylogenetically older and evolutionarily conserved role, the protein also catalyzes the terminal two steps during molybdenum cofactor (Moco) biosynthesis (Rajagopalan et al., 1982; Rajagopalan, 1985; Rajagopalan and Johnson, 1992), a critical anabolic process in almost all organisms. This metalloorganic cofactor, featuring a pyranopterin ligating a mononuclear molybdenum (Mo) ion, is the catalytically active component of almost all Mo-containing enzymes (reviewed in detail in Schwarz et al., 2009; Mendel, 2013), and is thus essential for cellular viability. Specifically, GephG adenylates the apo-form of the cofactor which is referred to as molybdopterin (MPT) resulting in the formation of adenylated MPT (AMP-MPT) (Kuper et al., 2004) in an ATP-dependent manner, and, subsequently, GephE deadenylates this reaction intermediate coupled to the insertion of the metal into the dithiolene moiety of the compound, thus forming the mature Moco (Kasaragod and Schindelin, 2016).

Dysfunctions with respect to gephyrin-mediated neurotransmission have been implicated in severe neurological disorders such as schizophrenia, autism, epilepsy, fragile X syndrome, Alzheimer’s disease, and also in hyperekplexia (Agarwal et al., 2008; Fang et al., 2011; Hales et al., 2013; Kratovac and Corbin, 2013; Dejanovic et al., 2014a, 2015). Any dysfunctionality with respect to the enzymatic function of gephyrin result in a rare, yet severe disease referred to as molybdenum cofactor deficiency, which manifests itself in severe abnormalities in neuronal functions and, ultimately, early childhood death (Reiss and Hahnewald, 2011).

In this review, we will focus on recent structural studies of the intact pentameric inhibitory neurotransmitter receptors and how these structures have helped us to better understand receptor architecture and shed light into the ligand–receptor interaction as well as the gating mechanisms of these receptors. In addition, we will also discuss how biochemical and structural studies of the gephyrin-GlyR-β interaction built a strong foundation to decipher the mechanism by which gephyrin aids in the alternate recruitment of GABA_A_Rs and GlyRs with these interactions taking place through a common universal receptor-binding pocket residing in the GephE domain.

## Structures of Glycine Receptors and Insights Into the Gating Mechanism

**FIGURE 2:**
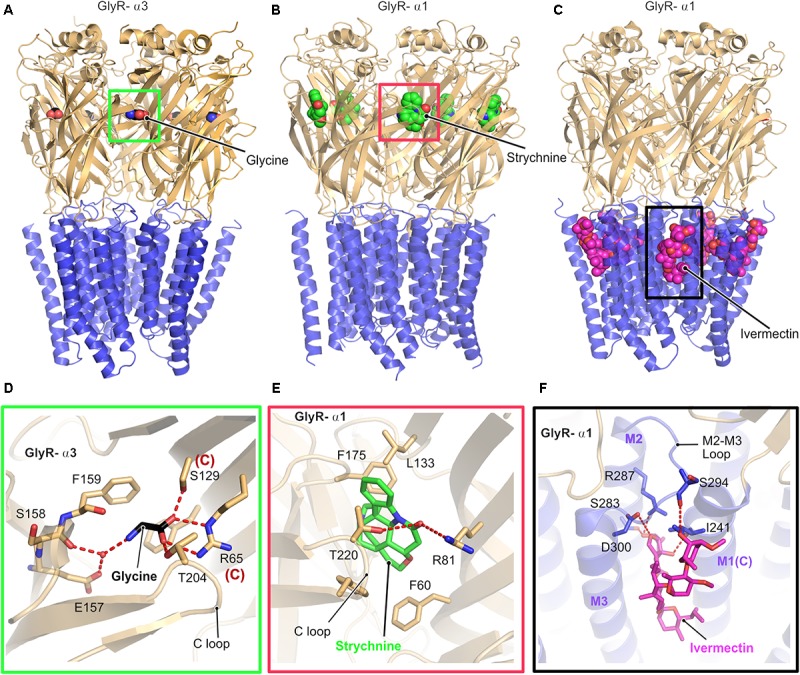
Architecture of glycine receptors. **(A–C)** Cartoon representation of the overall architecture of the homopentameric α3 GlyR in complex with glycine elucidated by X-ray crystallography (**A**, PDB: 5TIN) as well as the α1 GlyR in complex with strychnine (**B**, PDB: 3JAD) and ivermectin (**C**, PBD: 3JAF) by cryo-EM. The ECD is colored in light orange and the TMD in blue, bound ligands are shown in space filling representation. Enlarged views of the ligand binding pocket of the agonist glycine (black, **D**), the antagonist strychnine (green, **E**) and the positive allosteric modulator ivermectin (magenta, **F**). Bound ligands and critical residues which mediate the binding of the molecules are displayed in stick representation.

Glycine receptors are homo or heteropentameric receptors belonging to the Cys-loop superfamily of ligand-gated ion channels (pLGICs) (Lynch, 2004). Although biochemical and biophysical studies have helped us to understand the biological and electrophysiological properties of these receptors, a clear picture of the structure and gating mechanism came into existence only with the recent X-ray crystallographic and cryo-electron microscopy (Cryo-EM) structures (**Figure 2**) of the GlyR α3 and GlyR α1 homopentamers (Du et al., 2015; Huang et al., 2015). Du et al. (2015) solved the cryo-EM structures of the α1-homopentameric GlyR in complex with the antagonist strychnine, the agonist glycine and also the positive allosteric modulator ivermectin (**Figures 2B,C**). In contrast, the crystallographic structures of the GlyR α3-homopentamer bound to strychnine, glycine, ivermectin (Huang et al., 2017a) and also in complex with the analgesic potentiator (AM-3067) (Huang et al., 2017b) shed light into the gating mechanism of GlyRs composed of α3 subunits.

Although the cryo-EM structure was solved in the presence of glycine or in combination with glycine and ivermectin, the limited resolution of the structure did not allow to position the bound glycine. In contrast, a clearer picture of the interactions with the agonist glycine was derived from the crystal structure of the GlyR-α3 in complex with this agonist and analgesic potentiators. Both the GlyR α1 and α3 receptors share an identical architecture with a twisted β-sheet rich extracellular domain (ECD), composed of 10 β-strands, a transmembrane (TMD) domain composed of four α-helices and intracellular unstructured regions, which connect the transmembrane helices together with one extracellular loop (**Figure 2**). All structures share high structural similarity in their overall architecture with all known ligand gated ion channels (LICs) of the Cys loop superfamily such as the prokaryotic ELIC (Hilf and Dutzler, 2008) and GLIC (Nury et al., 2011) and a series of eukaryotic pLGICs including the *Caenorhabditis elegans* glutamate-gated chloride ion channel (GluCl) (Althoff et al., 2014), *Mus musculus* serotonin 5-HT3 receptor (Hassaine et al., 2014), and also the *Torpedo marmorata* acetyl choline receptor (AchR) (Miyazawa et al., 2003). A common denominator in all these structures is that the intracellular loop between transmembrane helices 3 and 4 (TM3 and TM4) has been replaced with a significantly shorter artificial sequence to facilitate the structural studies.

As proven by biochemical and physiological studies, both the antagonist strychnine and the agonist glycine occupy an overlapping binding site in the GlyR located at the interface between a principal and a complementary subunit (**Figures 2D,E**). A closer look at the structures and the binding pocket of the antagonist strychnine reveals that the binding of this alkaloid is mainly mediated by hydrophobic interactions with strychnine being stacked between aromatic residues with additional polar contacts from Arg81 of the complementary subunit and also Thr220 from the C-loop of the principal subunit of the receptor stabilizing the interaction (**Figure 2E**).

Inspection of the agonist-binding pocket reveals that glycine engages in a hydrogen-bonding network with a number of residues both from the principal and the complementary subunit. The ligand is mainly stabilized by the direct interaction of Thr204 residing in the C-loop of the receptor in the principal subunit and water-mediated contacts with the main chain of Ser158 and also Glu157 mediating the binding. In addition, Arg65 and Ser129 from the complementary subunit play critical roles in glycine binding through the formation of hydrogen bonds with the carboxylate of glycine.

The interaction with the positive allosteric modulator ivermectin involves a binding pocket which is situated close to the interface connecting the TMD to the ECD and is created by the M2 and M3 helices from the principal subunit and also the loop connecting these helices (**Figures 2C,F**). In addition, the complementary M1 helix also contributes significantly to the binding of ivermectin. The ivermectin binding pocket is structurally conserved beyond the GlyR as demonstrated by the crystal structure of the GluCl-ivermectin complex (Hibbs and Gouaux, 2011). The GlyR-bound ivermectin is stabilized by a hydrogen-bonding network and also multiple hydrophobic contacts contribute to this interaction. One of the crucial contacts with ivermectin involves a conserved arginine (Arg287 in GlyR-α1) which is located in the pore lining M2 helix of the receptor, thus imparting critical contributions to the gating mechanism of the receptors. The mutation of this Arg to either Gln or Leu has been identified in patients suffering from hyperekplexia and thus these structures explain the molecular basis of this disease (**Figure 2F**).

The comparison of all structures revealed that binding of the agonist alone or with the positive allosteric modulator ivermectin results in structural rearrangements, especially in the TM helix M2, which, in turn, facilitates pore opening. Hence, the receptor is in the fully open and hence active state when the agonist and allosteric modulator are bound as reflected in a distance of 10.6 Å between the Leu residues (Leu277) located at the 9′ position, compared to the glycine-only bound state with a pore diameter of 11 Å. Finally, the antagonist-bound structure exhibits a 9′ Leu-Leu distance of 8 Å, thus representing the closed conformation of the pore.

## Structures of Homopentameric and Chimeric GABA_A_ Receptors

**FIGURE 3:**
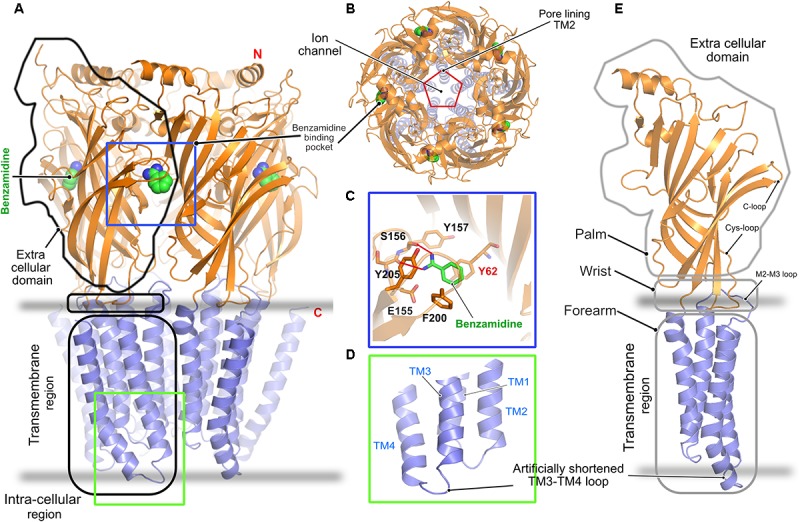
Architecture of the homopentameric GABA_A_R. **(A)** Crystal structure of the GABA_A_R β3 homopentamer in the desensitized state displayed in cartoon with the bound benzamidine in space filling representation (PDB: 4COF). **(B)** Top view of the receptor displaying the ion channel lining contributed by TM2. **(C)** Enlarged view of the agonist benzamidine (green) binding pocket where the bound ligand and crucial residues are shown in stick representation. **(D)** Enlarged view of the intracellular loops connecting the TM helices displaying the artificially shortened TM3-TM4 loop which aided in structure determination. **(E)** Cartoon representation of a GABA_A_R β3 monomer showing its relationship to a left arm.

In contrast to all other pLGICs, including the GlyRs, a clear understanding of the gating mechanism of the GABA_A_Rs is still lacking. The only crystal structure solved to date is that of the homopentameric GABA_A_R consisting of the β3-subunit (Miller and Aricescu, 2014). The crystal structure was solved in the presence of the protease inhibitor benzamidine, which turned out to be an agonist of the receptor (**Figure 3**). This structure, as in the case of the GlyRs, shares a high similarity with all known pLGICs as mentioned in the case of the GlyRs with a twisted β-sheet rich ECD, the TMD containing four α-helices and intracellularly located highly unstructured loop regions connecting these helices (**Figures 3A,E**). As in the case of the GlyR, the ion-channel pore is formed by the M2 helix of each subunit (**Figure 3B**). The binding of benzamidine is mediated by hydrophobic residues residing mainly in the principal subunit, as the compound is stacked in between two aromatic residues, Phe200 residing in the principal C-loop, and Tyr62 located in the complementary subunit (**Figure 3C**). Besides, a hydrogen-bonding network involving the ligand and Ser156 as well as Tyr157 contribute to this interaction. As with the GlyRs, structure determination was facilitated by substituting the long unstructured TM3-4 loop with a short artificial sequence (**Figure 3D**). While this first structure of a GABA_A_R provided important insights into the structural organization of the receptor, a clear understanding of its gating mechanism is still missing.

It has been known for a long time that the functions of multiple neurotransmitter receptors including the ionotropic GABA_A_Rs are modulated by neurosteroids (Belelli and Lambert, 2005; Hosie et al., 2006), and a recent study clearly demonstrated how the efficacy of GABA_A_Rs is modulated by these compounds (Mukherjee et al., 2017). Neurosteroids constitute a class of steroids, which are not only produced in the endocrine glands but can also be synthesized locally in the brain, and these compounds are crucial for the proper function of the brain. While some neurosteroids potentiate GABA_A_Rs such as the compounds pregnanolone and alphaxolone, other neurosteroids, such as sulfated and 3β-OH steroids, inhibit the same receptor, however, either mechanism of action remained elusive until recently (Wang, 2011).

**FIGURE 4:**
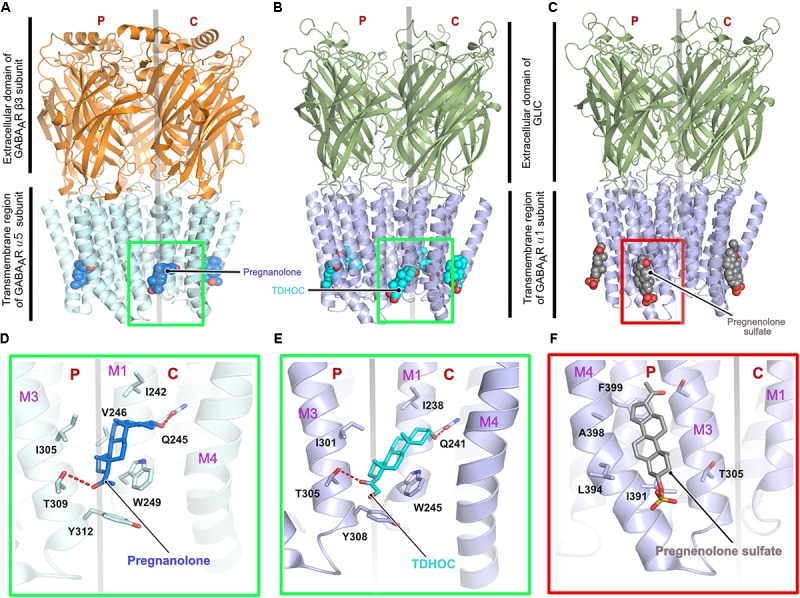
Architecture of the chimeric GABA_A_Rs. **(A)** Crystal structure of a chimeric GABA_A_R, resulting from the fusion of the transmembrane domain of the α5 subunit and the extracellular domain (ECD) of the β3 subunit, in complex with pregnanolone (PDB: 5O8F). Crystal structure of a chimeric GABA_A_R, resulting from the fusion of the transmembrane domain of the α1 subunit and the ECD of the prokaryotic GLIC, in complex with TDHOC (**B**, PDB: 5OSB) and pregnenolone sulfate (**C**, PDB: 5OSC). In all cases, the bound ligand is shown in space filling representation and the receptor as cartoon. Enlarged views of the ligand binding pocket of the potentiators, pregnanolone **(D)** and TDHOC **(E)** as well as the inhibitor pregnenolone sulfate **(F)**. Residues contributed by either the principal or complementary subunit are separated by a transparent gray line. The binding of the potentiators are mediated by the M3 helix from the principal and the M1 helix from the complementary subunit **(D,E)**, in contrast, binding of the inhibitory pregnenolone sulfate is mediated by helices M3 and M4 from the principal subunit **(F)**.

First insights into neurosteroid action at the molecular level were derived from chimeric crystal structures of GABA_A_Rs (Laverty et al., 2017; Miller et al., 2017) in complex with the potentiating neurosteroids pregnanolone and tetrahydro-deoxycorticosterone (THDOC) as well as the inhibitory steroid pregnenolone sulfate (**Figures 4A–C**). The chimeric versions of the GABA_A_Rs were either created by fusion of the transmembrane region of the α5 subunit fused to the ECD of the β3 subunit (Miller et al., 2017) or the transmembrane helices of the GABA_A_R α1 were fused to the ECD of the prokaryotic GLIC (Laverty et al., 2017). The neurosteroid-binding pocket resides further away from the interface between the TM helices and the extracellular region as seen for ivermectin in complex with either GlyR (Du et al., 2015) or the GluCl-channel (Hibbs and Gouaux, 2011; Althoff et al., 2014). The neurosteroids occupy a binding pocket in the transmembrane region, which is predominantly mediated by the principal M3 α-helix and complementary M1 α-helix of the receptors, irrespective of the downstream effect of the compounds. As expected, the binding interface is dominated by hydrophobic interactions, which are augmented by complementary interactions from either end of the potentiating neurosteroids mediated by putative hydrogen bonds from a conserved Thr (Thr309 in the α5 and Thr305 in the α1 subunit) residing in the M3 helix and another conserved residue (Gln245 in the α5 and Gln241 in the α1 subunit) located in the M1 helix of the α-subunits (**Figures 4D–F**). The structures also explain the specificity of these neurosteroids toward heteropentameric GABA_A_Rs, as the binding interaction has to be mediated by the obligatory β-α interface and can neither be provided by the α-β nor by the β-γ subunit interface. Although the δ subunit of the GABA_A_R contributes significantly to the potentiating effect of the neurosteroids (Vicini et al., 2002; Spigelman et al., 2003), a structural understanding of the mechanism of action through this subunit is still lacking. In contrast to the potentiators, inhibitory neurosteroid binding is solely mediated by the principal subunit of the receptor involving residues located in the M3 and M4 helices. The chimeric crystal structure of the GABA_A_R-GLIC in the presence of the inhibitory neurosteroid pregnenolone sulfate showed that these interactions are mainly mediated by the hydrophobic residues Ile391 and Phe399 of the M4 helix which sandwich the steroid in between them. In addition, bordering residues of the M3 helix also complement the interaction of pregnenolone sulfate with the receptor (**Figure 4F**).

## Structures of Heteropentameric GABA_A_ Receptors

**FIGURE 5:**
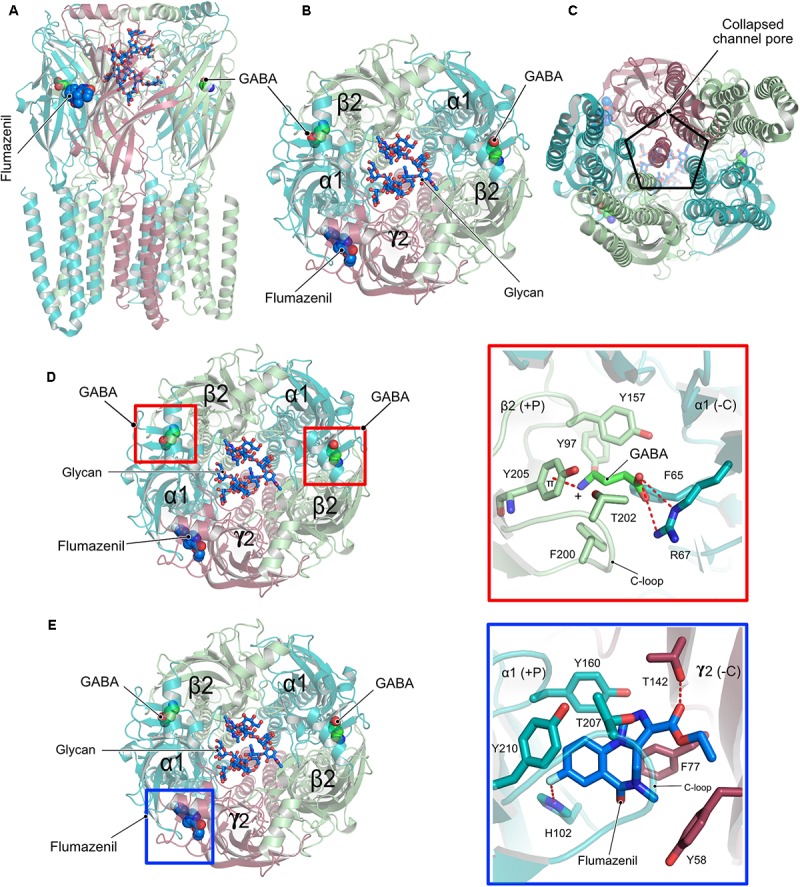
Structure and organization of a heteropentameric GABA_A_R. **(A)** Side view of the cryo-EM structure of the α1β2γ2 heteropentamer in complex with GABA and flumazenil shown in space-filling representation and with their C-atoms colored in green and blue, respectively. **(B)** View of the receptor from the ECD with the N-linked glycans (stick representation) of the α1-subunits pointing into the extracellular vestibule. **(C)** View of the receptor from the intracellular side clearly demonstrating how the γ2 subunit breaks the fivefold symmetry and obstructs the pore of the receptor. **(D)** Close-up view of the neurotransmitter binding pocket displaying the interaction of GABA and the β2-α1 receptor subunits. **(E)** Zoom into the benzodiazepam binding pocket displaying how flumazenil is bound at the α1-γ2 interface.

Although homopentameric and chimeric GABA_A_Rs provided critical insights into the overall organization of the receptor, ligand binding and the molecular basis for receptor potentiation by neurosteroids, the structures of the physiologically relevant heteropentameric receptors remained elusive until recently. Post-synaptically localized receptors are mainly composed of two α, two β and a γ subunit and a recent structure analysis of a heteropentameric (α1β2γ2) receptor in complex with GABA and flumazenil determined by cryo-EM (**Figures 5A–C**) shed light into some of the critical functional aspects of the structure and function of heteropentameric receptors (Zhu et al., 2018). This study reported two different structures of the heteropentamer, which differ in the conformation of the TMDs. A crucial feature of members of the Cys-loop family is that the channel pore is generated by the TM2 helix involving the participation of all five subunits of the pentameric functional receptor, however, the reported structures unexpectedly deviate from this classical element. A closer inspection of the published structures reveals that, although the ECD is structurally well-organized, the receptor pore has collapsed due to a movement of the γ2 TMD into the pore, thus eliminating the fivefold symmetry of the ion channel (**Figure 5C**). Whether this is an artifact of the structure solution or represents an inhibited state with physiological relevance remains unclear at present.

Besides the peculiar architecture of the receptor pore, the structures provided valuable insights into the interaction between the neurotransmitter GABA and the diazepam binding site antagonist flumazenil and the cognate receptor. The neurotransmitter occupies the canonical neurotransmitter binding site which is contributed by the β-α interface. The binding of GABA is mainly mediated by residues residing in the C-loop of the β-subunit via hydrophobic interactions. In addition, the carboxylate of GABA is involved in an extensive hydrogen-bonding network with Arg67 from the α-subunit while its positively charged amino group participates in a favorable cation-π interaction with Tyr205 of the α-subunit, thus additionally contributing to the stability of the neurotransmitter-receptor interaction (**Figure 5D**).

In contrast, flumazenil occupies the binding pocket created by the α-γ interface, which is created mostly by aromatic residues from both the α and γ subunits. This interaction is mediated by hydrophobic stacking interactions involving His102, Tyr160, and Tyr210 of the principal α subunit and another stacking interaction by Phe77 as well as a hydrogen bond with Thr142 from the complementary γ subunit.

Another highlight of the structure involves the structural characterization of the role of an N-linked glycan in the ECD of the α subunit, which is exclusive to heteromeric GABA_A_Rs. The bound glycan linked to Asn111 of the α subunits were well-defined in the structures and continuous density was observed for 5–8 monosaccharides with the two oligosaccharides from both α subunits pointing into the extracellular vestibule of the receptor. This glycosylation might in turn not only determine the stoichiometry of the α subunits in the heteropentamer but could also dictate the arrangement of the subunits within the pentamer (**Figures 5B,D,E**).

## Structure of the Moonlighting Protein Gephyrin

**FIGURE 6:**
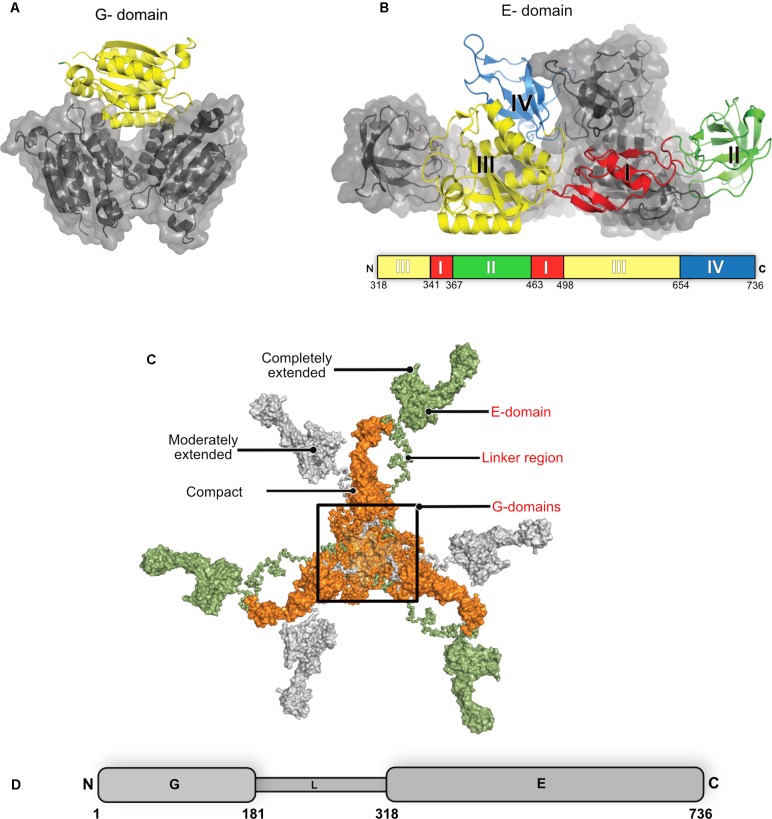
Structure of gephyrin. **(A)** Crystal structure of the N-terminal GephG trimer where one monomer is shown in cartoon representation (yellow) and the other two in surface representation in gray (PDB: 1JLJ). **(B)** Crystal structure of the C-terminal GephE dimer with one monomer colored according to its four subdomains (border lining residues of each subdomain are shown in the schematic diagram below the crystal structure) and the other monomer in surface representation in gray (PDB: 5ERQ). **(C)** Surface view of the ensemble of models of the full-length gephyrin derived from SAXS studies, where the compact state of the protein is represented in orange, the moderately extended state in gray and the fully extended state in green. **(D)** Schematic representation of the domain architecture of gephyrin.

The multi-domain inhibitory post-synaptic principal organizer gephyrin features two terminal domains connected by a ∼15 kDa large, highly unstructured linker region (**Figure 6**). In isolation, GephG (1–180) (**Figure 6A**), which is homologous to the bacterial MogA protein (Liu et al., 2000) and the plant Cnx1G domain (Schwarz et al., 2001; Sola et al., 2001), forms a trimer with a classical architecture of a Rossmann fold in each monomer. In contrast, the C-terminal GephE domains (318–736), which is evolutionarily related to the bacterial MoeA protein (Xiang et al., 2001) and plant Cnx1E domain (Krausze et al., 2017), forms a dimer (**Figure 6B**) (Kim et al., 2006; Kasaragod and Schindelin, 2016). GephE can be structurally subdivided into four subdomains (**Figure 6B**) with subdomain III sharing a similar architecture with the N-terminal GephG. Based on the oligomeric states of the terminal domains it has been proposed that the full-length protein forms a planar hexagonal scaffold, which provides anchoring points for the receptors on the membrane-proximal side and the ability to link to elements of the cytoskeleton on the opposite side (Kneussel and Betz, 2000; Xiang et al., 2001).

Full-length gephyrin has been recalcitrant toward crystallization until now, presumably owing to the highly unstructured and proteolytically sensitive linker region. The linker also contains multiple sites for post-translational modifications (PTMs) and mediates interactions with several partner proteins, namely microtubules, the dynein light chain (DLC) as well as peptidyl-prolyl isomerase NIMA-interacting protein 1 (Pin1) (**Figure 1**) (Ramming et al., 2000; Fuhrmann et al., 2002; Zita et al., 2007). In the absence of high resolution structural data, small angle X-ray scattering (SAXS) and atomic force microscopy (AFM) demonstrated that the full-length protein, after expression in *Escherichia coli*, is predominantly trimeric while, at the same time, being conformationally highly heterogeneous adopting compact as well as partially and extensively extended states (Sander et al., 2013). Strikingly, the full-length protein utilizes the trimer interface of GephG while the dimer interphase of the E domain is apparently masked due to still unknown reasons.

## Biochemical and Structural Basis for the Gephyrin-Receptor Interactions

**FIGURE 7:**
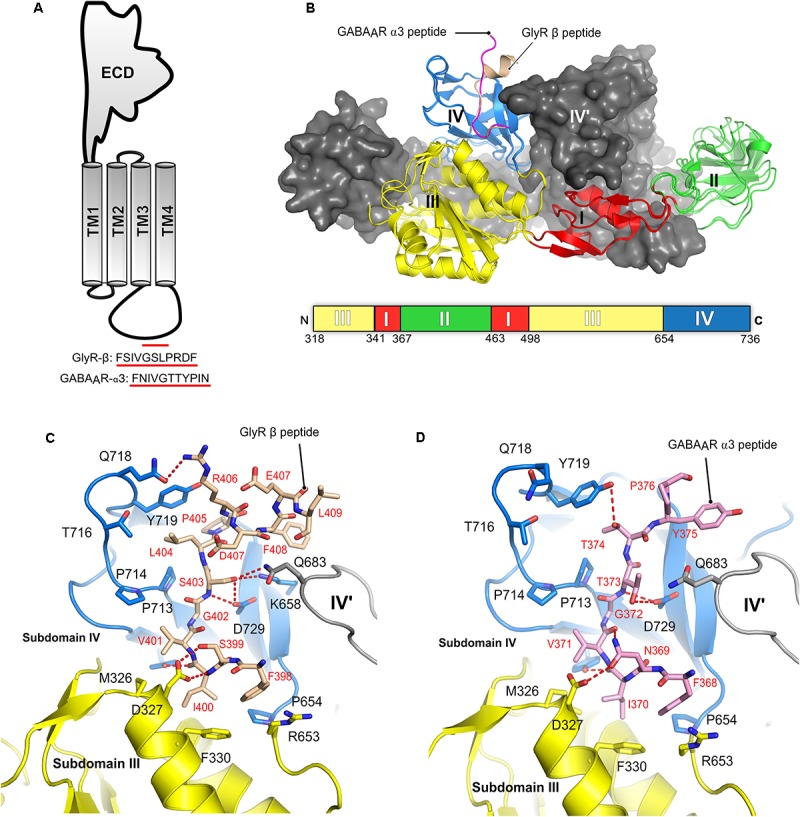
Structures of the gephyrin-receptor interactions. **(A)** Schematic representation of a single subunit of an inhibitory GABA_A_R or GlyR. The core binding motifs mediating the interaction of the receptor with gephyrin are underlined in red. **(B)** Overall architecture of the superimposed crystal structure of GephE in complex with the GlyR-β_49_ (PDB:2FTS) and GephE-GABA_A_R α3_11_ peptide (PDB: 4TK1), where the GephE subdomains are colored according to the scheme shown below the superimposed structure and are labeled with Roman numerals. Close-up view of the interaction of GlyR β_49_ (**C**, shown as light brown sticks) and GABA_A_R α3 (**D**, light pink sticks) with GephE. The receptor counterpart and residues of GephE, which are crucial for binding, are shown in stick representation and the others as a cartoon model. Please note that Asp327 of the E domain effectively interacts with the GlyR peptide forming both side chain-side chain and side chain-main chain interactions in contrast to the GABA_A_R α3 peptide and also the favorable interaction of Leu404 of the GlyR peptide in contrast to the partially polar Thr374 of GABA_A_R α3. These interaction primarily determine the different receptor affinities for GephE.

The interactions between gephyrin and the receptors are mediated by GephE and the large intracellular, highly unstructured loop region connecting transmembrane helices 3 and 4 (TM3-TM4) of the receptors (**Figure 7A**). Because of the highly unstructured nature of the intracellular loops, the structural basis for the Geph-receptor interaction remained enigmatic for a long time. Biophysical characterizations of the interaction between gephyrin and either the GlyR β-49 loop (residues 378–426) or shorter peptides derived thereof, ranging in length from 9 to 19 residues, indicated that the interaction is moderately strong with dissociation constants varying between the high nM to low μM range (Schrader et al., 2004; Kim et al., 2006; Maric et al., 2014a, 2015). In comparison, the TM3–TM4 loops of GABA_A_Rs interact with gephyrin with a lower affinity. Amongst the GABA_A_Rs, the α1 and α3 full-length TM3-TM4 loops bind with K_d_-values of 17 and 5.3 μM, respectively (Maric et al., 2011; Mukherjee et al., 2011; Tretter et al., 2011). When interpreting these dissociation constants, it should be kept in mind that under physiological conditions the encounter between the receptors and gephyrin is governed by two-dimensional diffusion as not only the receptors are located in the cell membrane but also gephyrin is recruited to the lipid bilayer. Furthermore, the oligomeric states of the receptors, which contain at least two gephyrin-binding subunits, and the oligomeric state of gephyrin will lead to avidity effects (see next section) which will significantly enhance these interactions.

Structural studies of the gephyrin-receptor interactions were mainly hindered due to the highly unstructured nature of the TM3–TM4 loop of the GlyRs and GABA_A_Rs. The first crystal structure of GephE in complex with a receptor-derived fragment was obtained in the presence of the GlyR β-49 loop. Although this structure provided a first glimpse of the Geph-receptor interaction, its moderate resolution of 3.25 Å made mapping of the residues mediating the interaction impossible (Sola et al., 2004). In the GephE dimer only one monomer was found to interact with the ligand and here only a short peptide of five residues (modeled as poly-alanine due to the poor electron density) could be visualized. A subsequent crystal structure at 2.4 Å resolution (Kim et al., 2006) of the GephE-GlyR-β49 complex mapped the interactions in both subunits of the complex (**Figures 6C**, **7B**). Although the crystallization was carried out with a 49-residue long fragment, only 13 residues (^398^Phe-Leu^410^) could be resolved in the crystal structure. Subsequently, crystal structures of GephE in complex with GABA_A_R α3 (2.7 Å) (Maric et al., 2014a) derived peptides described the interactions of gephyrin with the highest affinity subunit of the GABA_A_R (Maric et al., 2011) in atomic detail (**Figure 7D**).

Collectively these structures showed that the receptor occupies a primarily hydrophobic binding pocket created mainly by subdomain IV and partially by subdomain III of GephE. Both receptors were found to interact in a nearly identical manner at the N-terminal ends of the core binding motifs while they differ substantially at their C-termini where the interactions become receptor specific. In case of the GlyR β subunit the peptide adopts an overall arrangement mimicking the letter “C” with a short 3_10_ helix at the C-terminal end (**Figure 7B**) of the core binding region, while the GABA_A_R α3 -subunit derived peptide, in comparison, adopts a straighter trajectory in the binding pocket resembling the letter “L” in its main chain conformation (**Figure 7B**) (Kim et al., 2006; Maric et al., 2014a,b).

The N-terminal three residues of both receptors (^398^FSI^400^ in GlyR β and^368^FNI^370^ in GABA_A_R α3) (**Figures 7C,D**) interact with GephE in a virtually identical manner, while receptor specific interactions involve residues at the C-termini of the core binding motifs. In both cases the primary determinant of the interaction is the stacking of Phe330 located in subdomain III of GephE with Phe398 and Phe368 of the GlyR β and GABA_A_R α3 subunits, respectively. Two critical residues which determine receptor specific interaction are Ser399 of GlyR β/Asn369 of GABA_A_R α3 at the N-terminal end and the C-terminally located Leu404 of GlyR-β/Thr374 of GABA_A_R α3. Ser399 of GlyR β effectively interacts with the side chain of Asp327 and, at the same time, stabilizes the GephE-GlyR interaction by forming a side chain-main chain interaction with Val401 of the GlyR β subunit (**Figure 7C**). In comparison, Asn369 of GABA_A_R α3 cannot satisfy both of these requirements and hence is in part responsible for the decrease in affinity (**Figure 7D**, see also the section on domain swapped crystal structures of GephE in the following paragraph). An even more critical determinant of the higher affinity of the GlyR β-subunit toward gephyrin is Leu404. The hydrophobic Leu404 effectively interacts with a GephE pocket enriched in hydrophobic residues, which is less suited to accommodate the partially polar side chain of Thr374 from the GABA_A_R α3 subunit, thus also affecting the affinity of the GABA_A_R α3 to gephyrin.

The crystal structures were validated by mutational and biophysical analyses through isothermal titration calorimetry (ITC). In particular, a chimeric peptide derived from the GlyR β-subunit in which residues Ser399/Leu404 were exchanged to Asn/Thr as present in the GABA_A_R α3 subunit resulted in an affinity, which was more similar to the WT-GABA_A_R α3 (K_d_ = 180 μM). In contrast, the chimeric peptide derived from the GABA_A_R α3 subunit with the reciprocal exchange of Asn369/Thr374 to Ser/Leu as found in the GlyR β-subunit exhibited an affinity which was similar to that of the WT-GlyR-β to GephE (K_d_ = 8 μM). Once again, this mapped and confirmed the primary determinants of the receptor specific interaction with gephyrin (Maric et al., 2014a).

Comparison of the sequences of the core binding regions of the gephyrin-interacting part of the receptors revealed that there are multiple common denominators throughout the receptor binding regions. Critically, a conserved hydrophobic residue, Phe398 of the GlyR β and Tyr340, Tyr339 as well as Phe368 of the GABA_A_R α1-3 subunits, respectively, plays a critical role (**Table 1**). The cocrystal structures of peptides derived from the GlyR β and the GABA_A_R α3 subunits demonstrated that Phe398/Phe368 engage in a critical hydrophobic stacking interaction with Phe330 of GephE as mentioned above. Mutation of either Phe330 of gephyrin or the aromatic residue in the receptor (Phe to Ala or Tyr to Ala mutations, respectively) completely abolished the gephyrin-receptor interaction as measured by ITC (**Table 1**). On the other hand, the conserved tyrosine in the GABA_A_R α1-3 subunits serves as a second denominator amongst the GABA_A_Rs. Mutation of this residue was also shown to impact the gephyrin-receptor interaction (Kim et al., 2006). Comparing the GlyR β and GABA_A_R α3 derived peptides on a structural level revealed that Phe408 of the GlyR β-subunit is located in the same binding pocket as Tyr375 of the GABA_A_R α3 subunit, although these residues are located in the structurally divergent C-terminal ends of the peptides and also do not align when the sequences are compared (**Figures 7C,D**) Although the crystal structures of GephE in complex with peptides derived from other synaptically localized α-subunits of the GABA_A_Rs are still missing, the existing crystal structure of the GephE-GABA_A_R α3 complex allows one to model the GephE-GABA_A_R α1 or α2 interactions with reasonable accuracy.

**Table 1:** Core binding motifs of inhibitory neurotransmitter receptors which interact with gephyrin.

Receptor	Subunit	Core binding motifs
GlyR	**β^#^**	**^398^F**SIVGSLPRD**F^408^**
GABA_A_Rs	**α1**	**^340^Y**APTATS**Y**TPN**^350^**
	**α2**	**^339^Y**AVAVAN**Y**APN**^349^**
	**α3^#^**	**^368^F**NIVGTT**Y**PIN**^378^**
	**β2**	AGLPRHSFGRNALERHVAQKKSRL
	**β3**	QSMPKEGHGRYMGDRSIPHKKTHL

*The aromatic residue at the N-terminus of the core binding motif serves as the first common denominator while the second aromatic side chain Y/F at the C-terminus, although not conserved in sequence, nevertheless occupies the same binding pocket in the crystal structure and hence acts as the second anchor point in these motifs. ^#^ Structurally validated by X-ray crystallography*.

In contrast to the α subunits, the β2 and β3 subunits do not share a common denominator, neither with the α-subunits nor between them (Kowalczyk et al., 2013), hence a full atomic level understanding of the subunit specific interactions of gephyrin amongst the entirety of the GABA_A_Rs still remains elusive (**Table 1**).

## Modulation of Gephyrin-Receptor Affinity

In the physiological context of inhibitory post-synapses one must take into consideration that there are multiple pentameric receptors available in close proximity to gephyrin, which could potentially regulate the interaction between gephyrin and post-synaptic receptors, or, in other words, avidity effects will most certainly play a crucial role. Thus, to take this into consideration Maric et al. (2014b, 2015) synthesized and performed ITC experiments with gephyrin and dimerized receptor peptides. The dimerization of the GlyR β and GABA_A_R α3 peptides were carried out with five different cross-linkers. These studies revealed that the length of the peptides and also the type of crosslinker had an impact on the affinity between gephyrin and inhibitory receptors. The results displayed a remarkable increase in the affinity of the gephyrin-receptor interaction up to a 1200-fold enhancement for GlyR β derived peptides and up to 800-fold for GABA_A_R α3 derived peptides (Maric et al., 2014b, 2015). In addition to achieving an affinity potentiation, the dimerized versions of the peptides helped to decipher gephyrin-receptor interactions at high resolution. Crystal structures of GephE were determined in complex with two dimerized versions of the receptor derived peptides: (i) GephE in complex with a decameric GlyR β peptide crosslinked with a para-phenyl-based crosslinker and (ii) GephE in complex with a nonameric GABA_A_R α3 peptide crosslinked with a polyethylene glycol (PEG) derivative. Although GephE-peptide complexes were solved in a domain-swapped arrangement, the structures mapped the interaction at near atomic resolution (2 Å).

**FIGURE 8:**
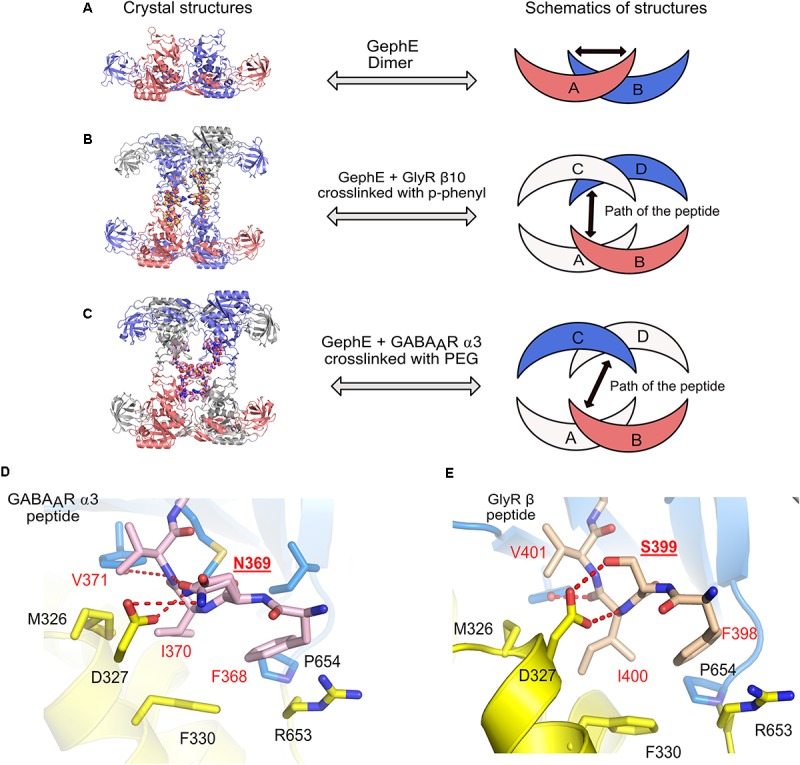
Domain swapped crystal structures of GephE-receptor derived peptide complexes. **(A)** Crystal structure of the GephE dimer in the apo form. Crystal structures of domain swapped GephE-peptide complexes determined in the presence of high affinity dimeric peptide derived from either GlyR β (**B**, PDB: 4U91) or GABA_A_R α3 (**C**, PDB: 4U90) subunits. The peptide takes a straight trajectory in case of the GlyR-β peptide in contrast to the GABA_A_R α3 peptide which adopts a cross trajectory to connect two GephE monomers. **(D,E)** Enlarged views of the N-terminal region of the GABA_A_R α3 and GlyR-β core binding motifs. Please note that in the case of the GABA_A_R α3 peptide **(D)** Asn369 (underlined label) is forced to exhibit dual conformations of its side chain to match the hydrogen bonding potentials of its partners, in contrast, a single conformation of Ser399 in GlyR-β effectively stabilizes the GephE-GlyR interaction.

Three-dimensional (3D) domain swapping is a common phenomenon which is observed with many proteins and can be defined as a mechanism by which two or more protein molecules form a dimer or higher order oligomer by exchanging an identical structural element, which, in turn, mediates oligomerization of the proteins (Liu and Eisenberg, 2002). One of the classical examples of 3D domain swapping is the anti-apoptotic protein BCL-X_L_ where the hinge loop region between α-helices five and six transforms and fuses the two α-helices, thus mediating domain swapping and subsequently also dimerization of the protein (O’Neill et al., 2006). In case of the domain swapped structures of GephE in complex with the receptor derived peptides (**Figures 8A–C**), the cross-linker region of the dimeric peptide acts as the artificial hinge region, thus mediating domain swapping in 3D. Interestingly the two structures revealed two different domain swapped arrangements in which two adjacent GephE dimers are connected. In the GephE-GlyR β structure, the peptide takes a straight trajectory (**Figure 8B**), while in the GephE-GABA_A_R α3 complex, the peptide displays a cross trajectory (**Figure 8C**). Despite domain swapping, especially the GephE-GABA_A_R α3 structure unveiled additional critical information with respect to the role of Asn369 of the GABA_A_R α3. The structure showed that this residue adopts two different conformations, which modulate the interaction with Asp327 of GephE (**Figure 8D**) (Maric et al., 2015), thus also explaining the relatively poor affinity of the GABA_A_R in comparison to the GlyR β (**Figure 8E**). Moreover, systematic variations of receptor-derived peptides allowed for the successful mapping of residues, which are of critical importance for binding. Finally, labeling of engineered high affinity peptides with small organic fluorophores was also successfully utilized as a powerful tool for the specific visualization of inhibitory post-synaptic organizations (Maric et al., 2017). The concept of peptide dimerization can potentially be extended toward other subunits of the GABA_A_R family such as α1 and α2, which display even lower affinity toward gephyrin (Maric et al., 2011) and hence this strategy could ultimately help us to understand the subunit specific interactions between gephyrin and GABA_A_Rs.

## Thermodynamic Parameters and Possible Existence of a Second Receptor Binding Site

Although all crystal structures of GephE in complex with the core binding region of the receptor derived peptides have shown that the structure has a stoichiometry of 1:1, it is still a point of dispute whether the gephyrin-receptor interactions, in particular that involving the GlyR β-subunit, are mediated by two binding sites, i.e., a high affinity and a second low affinity binding site (Schrader et al., 2004; Kim et al., 2006; Herweg and Schwarz, 2012; Dejanovic et al., 2015; Grunewald et al., 2018). The existence of the second low affinity binding site was initially proposed by Sola et al. (2004) based on their crystal structure and the observed differences in GlyR peptide binding in the two GephE monomers which, as stated above, resulted in one occupied and one empty binding receptor binding pocket. Although there are no obvious differences in the receptor-binding pocket of both monomers of GephE, multiple studies employing ITC supported the argument of two binding sites with different affinities as summarized in **Table 2**. In contrast to the first crystal structure, all of these studies were interpreted in such a way that the high affinity binding site corresponds to the symmetrical GephE-GlyR β interaction visualized subsequently by Kim et al. (2006) while the existence of a second binding site involving residues within the β49 construct but outside the core binding site (residues 398–410) and a different binding pocket located in GephE was inferred. In support of this assumption ITC studies involving short chemically synthesized peptides, either in the monomeric or dimeric form, consistently yielded a single binding site, while only studies with the longer β49 construct yielded two binding sites. Nevertheless, it cannot be ruled out that additional factors such as impurities/degradation of the recombinantly produced β49 fragment, phosphorylation by the *E. coli* host or conformational heterogeneity (i.e., a mixture of different conformers or oligomers of full-length gephyrin) give rise to the additional binding isotherms. This assumption is supported by the sub-stoichiometric n-values, in particular that of the high affinity binding site which is typically estimated to be in the 0.2 to 0.3 range, with the low affinity site also being sub-stoichiometric with occupancies between 0.5 and 0.6. In contrast to the Geph-GlyR interaction, all ITC studies investigating the Geph-GABA_A_R interactions have been interpreted with a one site binding model, although GABA_A_Rs are dependent on the same binding site as stated above.

**Table 2:** Summary of thermodynamic parameters of the gephyrin-GlyR β interaction.

Construct	Ligand	Model	K_D1_ (μM)	n_1_	K_D2_ (μM)	n_2_	Reference
GephFL	β-49	2-site	0.4	0.98	30	0.83	Schrader et al., 2004
GephE	β-49	2-site	0.2	0.60	11	0.51	
GephFL	β-49	2-site	0.09	0.35	15.6	0.45	Kim et al., 2006
GephE	β-49	2-site	0.12	0.64	7.8	0.65	
GephFL	β-long	2-site	0.14	0.65	7.7	0.6	Mukherjee et al., 2011
GephFL	β-49	2-site	0.02	0.28	2.9	0.57	Specht et al., 2011
GephE	β-14	1-site	4.9		-	-	Maric et al., 2011
FL (Trimer from *E. coli*)	β-49	2-site	0.05	0.29	6.3	0.55	Herweg and Schwarz, 2012
FL (Hexamer from insect cells)	β-49	1-site	0.4	0.22	-	-	
GephE	β-14	1-site	6.3	0.98	-	-	Maric et al., 2014a,b
GephE	β-19	1-site	2.8	0.96	-	-	
GephE	β-49	1-site	2.1	0.91	-	-	
GephFL	β-19	1-site	2.5	0.88	-	-	
GephFL	β-49	1-site	2.4	0.83	-	-	
GephFL	β-49	2-site	0.02	0.28	2.93	0.57	Dejanovic et al., 2015
GephFL G375D	β-49	1-site	0.39	0.20	-	-	

## Post-Translational Modifications of Gephyrin

**FIGURE 9:**
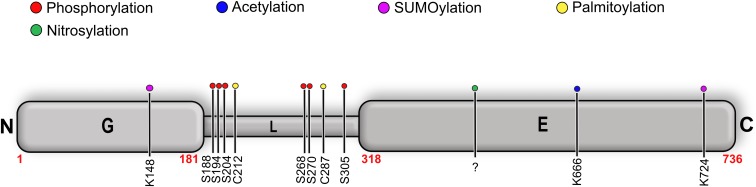
Post-translational modifications (PTMs) of gephyrin. Schematic representation of well-characterized PTMs of gephyrin where the different PTMs are colored according to the accompanying scheme. Location of the PTM as indicated by corresponding residue number and type.

Gephyrin was shown to undergo extensive PTMs including phosphorylation (Langosch et al., 1992; Tyagarajan et al., 2011; Herweg and Schwarz, 2012; Flores et al., 2015), palmitoylation (Dejanovic et al., 2014b), acetylation, SUMOylation (Ghosh et al., 2016), and nitrosylation (Dejanovic and Schwarz, 2014) (**Figure 9** and **Table 3**). The sites for the PTMs have been predominantly mapped to the linker region, which may have critical implications for its structure and also that of the full-length protein as well as on downstream signaling pathways. Most phosphorylations result in an upregulation of gephyrin clustering (Zacchi et al., 2014), e.g., Calcium/calmodulin-dependent protein kinase II (CaMKII) dependent phosphorylation of Ser305 promotes the formation of inhibitory synapses (Flores et al., 2015). In contrast, cross-talk phosphorylations of Ser268 and Ser270 by the kinases ERK1/2 and GSK3β, respectively, mark gephyrin for calpain-dependent degradation (Tyagarajan et al., 2011, 2013). Phosphorylation of Ser188, Ser194 and Ser200, residues located at the beginning of the linker region, acts as obligatory marker for the recruitment of Pin1 (Zita et al., 2007). The resulting gephyrin-Pin1 interaction has been implicated in conformational changes in gephyrin, which potentially positively modulate the gephyrin-GlyR interaction. In addition to the interaction with gephyrin, the important cell adhesion molecule neuroligin also has been shown to be a substrate of Pin1 where this interaction in turn results in a downregulation of GABAergic inhibitory synapse formation (Antonelli et al., 2014) (**Figure 9**).

**Table 3:** Summary of well-characterized post-translational modifications of gephyrin and their consequences for inhibitory post-synaptic functionality.

Number	Type of PTM	Location in Geph	Residue number/s	Consequences	Reference
1	Phosphorylation	Linker	Ser188, Ser194, and Ser200	Pin-1 recruitment and modulation of gephyrin-GlyR interaction	Zita et al., 2007
2	Phosphorylation	Linker	Ser268 and Ser270	Crosstalk of phosphorylation which acts as the marker for gephyrin degradation by calpain	Tyagarajan et al., 2011, 2013
3	Phosphorylation	Linker	Ser305	Upregulation of gephyrin clusters and potentiation of GABAergic currents	Flores et al., 2015
4	*S*-Nitrosylation	GephE	Residue not mapped	Downregulation of size of gephyrin clusters	Dejanovic and Schwarz, 2014
5	Palmitoylation	Linker	Cys212 and Cys287	Positive effect on gephyrin clustering and potentiation of GABAergic currents	Dejanovic et al., 2014b

Palmitoylations of Cys212 and Cys284 by the palmitoyl transferase DHHC-12 have a positive effect on gephyrin clustering and also a potentiating effect on GABAergic neurotransmission (Dejanovic et al., 2014b). In contrast to all linker-associated PTMs, S-nitrosylation has been mapped to the E-domain, where overexpression of the neuron-specific nitric oxide synthase resulted in a decreased cluster size of gephyrin in primary hippocampal neurons (Dejanovic and Schwarz, 2014). SUMOylation of GephG on Lys148 and of GephE on Lys724 has been speculated to act upstream of the phosphorylation of Ser268 and the acetylation of Lys666 (Ghosh et al., 2016). This study showed that de-conjugation of either of these residues manifests itself in the deacetylation of Lys666 and dephosphorylation of Ser268, which, in turn, promotes gephyrin clustering.

In addition to the aforementioned PTMs of gephyrin, its interacting receptors are also subject to PTMs. The most striking of these is the phosphorylation of Ser403 of the GlyR β-subunit, which resides in the core binding motif and mediates the gephyrin-GlyR β interaction. Ser403 is phosphorylated in a protein kinase C dependent manner, which, in turn, downregulates the gephyrin-GlyR β interaction. In summary, all of the aforementioned PTMs play a critical role in the gephyrin-mediated formation, maintenance and plasticity of inhibitory post-synapses (Specht et al., 2011). In addition, Thr375 of the GABA_A_R α1 has also been proposed as a putative phosphorylation site and ITC experiments with the phosphomimetic mutant (T375E/D) has shown that the modification causes a down regulation of the gephyrin-GABA_A_R α1 interaction (Mukherjee et al., 2011).

## An Additional Layer of Complexity: Alternative Splicing of Gephyrin

**FIGURE 10:**
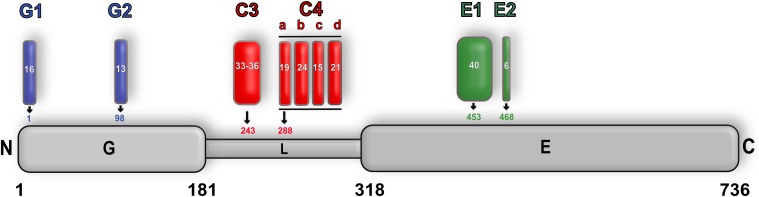
Alternative splicing of gephyrin. Schematic representation of alternative splicing of gephyrin with GephG specific cassettes colored in blue, linker cassettes in red and E domain cassettes in green along with the length of the cassettes and also the insertion site is shown for each cassette. The full-length protein is numbered according to the P1 splice variant nomenclature.

One additional layer of complexity regarding the function of gephyrin is the existence of alternative splicing (Prior et al., 1992; Paarmann et al., 2006a,b; Herweg and Schwarz, 2012). The *GPHN* gene is a highly mosaic gene encoded on chromosome 14 (q23.3) consisting of 30 exons, of which nine have been shown to undergo alternative splicing (**Figure 10**). The resulting differences in primary sequence of the protein in turn modulate the structure as well as sub-cellular localization of gephyrin and thus both the neurotransmitter receptor anchoring and molybdenum cofactor biosynthetic functions of gephyrin. This is summarized in detail (Fritschy et al., 2008) including a new nomenclature for the splice variants. Most additions or modifications of the exons affect the linker region (five cassettes) and mainly influence the clustering behavior of gephyrin. Insertion of the G2 cassette (previously known as the C5 or C5′ cassette) into GephG results in an altered oligomeric state of this domain and, in turn, not only abolishes the Moco-biosynthetic function of gephyrin (Smolinsky et al., 2008) but also prevents the co-localization of gephyrin with the GlyR β subunit (Saiyed et al., 2007) (**Figure 10**). In addition, a variant containing the C3 splice cassette modulates the gephyrin-GlyR β interaction by reducing the affinity 10-fold in comparison to the corresponding C4-containing variant after expression of both variants in Sf9 cells (Herweg and Schwarz, 2012).

## Gephyrin Interaction Partners and Effects of These Interactions on Post-Synaptic Organization

Besides the direct interaction with inhibitory neurotransmitter receptors, gephyrin also interacts with a fairly extended repertoire of macromolecules (reviewed in detail in Tyagarajan and Fritschy, 2014; Choii and Ko, 2015; Uezu et al., 2016) (**Figure 1**), thus acting as the principal organizer in maintaining the normal functionality of the majority of inhibitory post-synapses. Gephyrin was shown to interact with microtubules, however, as post-synaptic sites are rich in actin filaments, the physiological relevance of this interaction remains unclear. Since the linker region (exon 14) of gephyrin shares high sequence identity with the classical microtubule binder tau, it was proposed to mediate the gephyrin-tubulin interaction (Ramming et al., 2000). Physiologically more relevant is the fact that gephyrin acts as cargo protein for the tubulin-driven motor protein subunits KIF5 through simultaneous binding to heteropentameric GlyRs which mediates the recruitment of these receptors to the synaptic membrane (Maas et al., 2006, 2009). Again, the molecular details of these interactions have yet to be deciphered. Although the transport mechanisms of GABA_A_Rs are less understood, it has been shown that KIF5 also plays a crucial role in GABA_A_R transport to the synaptic membrane by its association with the huntingtin associated protein 1 (HAP1) (Twelvetrees et al., 2010) and also with the GABA_A_R associated protein (GABARAP) (Nakajima et al., 2012).

Dynein light chains 1 and 2 (DLC1 and DLC2) have also been identified to interact with gephyrin. DLC1 and 2 are components of the dynein motor, which helps in the transport of various cargos. The interaction between DLC and gephyrin is mediated by residues 181–243 near the N-terminal end of the linker region of gephyrin (Fuhrmann et al., 2002). Although these residues were shown to be sufficient to mediate the direct interaction between gephyrin and DLC, the implication of this interaction remains to be elucidated.

In addition, gephyrin also interacts with the other major cytoskeletal element, actin filaments, however, in contrast to microtubules no direct interaction with actin has been reported. Instead the interaction is accomplished via actin-associated proteins such as Profilin and members of the Mena/Vasp family, with the latter interaction being mediated by GephE. In addition, gephyrin competes with G actin and also phospholipids for an overlapping binding site on profilin, thus acting as a bridge between gephyrin and microfilament systems (Giesemann et al., 2003).

Neuroligins (NLs) were among the first cell adhesion molecules being identified in neuronal cells and include four family members (NL1–NL4). NL2 has been shown to be localized at GABAergic inhibitory post-synapses (Dong et al., 2007) whereas NL4 is exclusively present at glycinergic post-synapses (Hoon et al., 2011). NL2 interacts with presynaptic neurexins, another family of cell adhesion molecules, to form a trans-synaptic complex, thus bridging the presynapse to its post-synaptic counterpart. In contrast, NL3 has been shown to be localized at glutamatergic and also GABAergic synapses. Furthermore, NL3 has also been shown to interact with NL1 as well as NL2, although the functional implications of this complex formation have not been established (Budreck and Scheiffele, 2007). Amongst the four family members, NL2 is the only protein that has been shown to interact with gephyrin. This interaction is mediated by the unstructured C-terminal region of NL2 and GephE (Poulopoulos et al., 2009). Moreover, NL2 plays a crucial role in the collybistin-mediated targeting of GABA_A_Rs and gephyrin to the post-synaptic membrane (Soykan et al., 2014).

Gephyrin also directly interacts with the aforementioned guanine nucleotide exchange factor (GEF) collybistin (CB) (Kins et al., 2000). This GEF, which is also referred to also as Arhgef9, belongs to the DBL superfamily of exchange factors. Structurally, CB features an N-terminal Src-homology domain 3 (SH3) followed by a DBL homology (DH) and a pleckstrin homology (PH) domain (Harvey et al., 2004; Fritschy et al., 2012). CB has been shown to undergo alternative splicing, in which the spliced variant differs with respect to presence of the SH3 domain and also exhibit variable lengths of the translated protein at the C-terminus. The SH3 domain has been shown to play a crucial role in modulating the activity of the protein. Formation of the Geph-CB complex is mediated through GephE and the DH domain of CB, and this interaction is crucial during early stages of neuronal development and is critical for the targeting of gephyrin to the post-synaptic membrane (Grosskreutz et al., 2001). In addition to the interaction with gephyrin, the active state of CB has also been shown to directly interact with the GABA_A_R α2 subunit which targets the receptor to the post-synaptic membrane (Soykan et al., 2014) (**Figure 1**). Confirming this result, a recent study also proved the direct and preferential binding of CB (mediated by the SH3 domain) to the GABA_A_R α2 (Hines et al., 2018). It has been shown that receptor binding and the CB binding sites on gephyrin are located in close proximity in the primary sequence. In addition, gephyrin and CB were also proposed to rely on the same binding site on the GABA_A_R α2 subunit, which could ultimately result in a tripartite interaction of these proteins (Saiepour et al., 2010).

Most of the interactions gephyrin engages in are mediated by either the linker or GephE. Quite recently, IQSEC3, a member of the ADP ribosylation factor-guanine nucleotide exchange factor (ARF-GEF) family was shown to also interact with gephyrin (Um et al., 2016). Structurally, IQSEC family members contain coiled coil (CC) and IQ domains at the N-terminus followed by a SEC domain and a PH domain at the C-terminus. The three members of the IQSEC family, although being closely related in their domain organization, differ with respect to their cellular localization. IQSEC1 and 2 have been found to be exclusively localized to excitatory post-synapses, while IQSEC3 is present at inhibitory post-synapses (Um, 2017). Quite interestingly, the interaction between gephyrin and IQSEC3 is meditated by GephG, thus being the first interaction partner of gephyrin to engage its N-terminal domain (Um et al., 2016). Although the molecular basis of the interaction remains to be deciphered, it would provide an interaction partner, which could potentially modulate the oligomeric state of the trimeric gephyrin (**Figure 1**).

## Future Perspectives and Outlook

Biochemical, electrophysiological and, in particular, recent structural studies have shed light on the overall architecture and also the gating mechanism of inhibitory neurotransmitter receptors. Although homomeric and chimeric structures of the GlyRs or GABA_A_Rs provided important insights into the architecture and also some of the functional aspects of the receptors, a thorough understanding of the heteromeric receptor of both of these family members was missing until very recently. The first structure of a heteropentameric GABA_A_R (Zhu et al., 2018), despite exhibiting an unsual architecture in the transmembrane region, provided additional insights into ligand binding and the role of N-linked glycans in receptor assembly. Future research effort will undoubtedly address GABA_A_R assemblies and potential receptor asymmetry. At the same time numerous crystal structures of GephE in complex with peptides derived from either the GlyRs or GABA_A_Rs have helped us to understand the molecular mechanism of the alternative receptor-specific recruitment and clustering of these inhibitory receptors by gephyrin. Nonetheless, in addition to the interaction with the GABA_A_R α3, gephyrin also interacts with the α1 and α2 as well as possibly the β2-3 subunits of GABA_A_Rs and future crystal structures of GephE in complex with peptides derived from these subunits would further strengthen our understanding about the subunit-specific interaction of gephyrin with different GABA_A_Rs. The concept of dimerization (Maric et al., 2015) could be applied to the other subunits of the receptor subunits which would strengthen the affinity between the GABA_A_Rs subunits (α1-2 and β2-3) and gephyrin due to the avidity effect and aid in their structural characterization.

As of now a high-resolution structure of full-length gephyrin is still missing and hence the dynamics of the interaction of gephyrin with the receptors still remain elusive. Depiction of a high resolution structure of either mammalian gephyrin or any homolog of gephyrin, will also clarify the enigmatic oligomeric organization of gephyrin and thus the overall architecture of inhibitory post-synaptic specializations. It should also be taken into consideration that all receptor structures derived so far were achieved by shortening the large intracellular loop connecting TM3 and TM4, which is critical for the interaction of both receptor types with gephyrin.

In addition to gephyrin, other proteins such as collybistin (Soykan et al., 2014) and the cell adhesion molecule NL2 also influence receptor organization and thus the architecture of inhibitory post-synapses. Hence, future research should also be directed toward the structural elucidation of the intact receptor in complex with its interaction partners as extensively performed with the α-amino-3-hydroxy-5-methyl-4-isoxazole propionic acid (AMPA) subtypes of the iGluRs in complex with auxiliary γ-subunits (Chen et al., 2017; Twomey et al., 2017). These studies helped us to understand the dynamics of these receptors beyond the level of a single receptor. In addition, the structural elucidation of the iGluRs with trans-synaptic organizers have also strengthened our understanding of the supramolecular organization at excitatory synapses (Elegheert et al., 2016). Similar structural approaches can also be envisioned in the case of the GABA_A_Rs. Here the newly discovered auxiliary subunit GARLH (Davenport et al., 2017; Yamasaki et al., 2017) has been shown to play a crucial role in the assembly of supramolecular post-synaptic complexes by its association with both GABA_A_R and NLs, thus impacting the assembly and functionality of the receptor.

Multiple small molecule modulators of neurotransmission have been discovered so far and most of these compounds directly bind to the neurotransmitter receptors, thus fine-tuning the gating properties of the receptors and ultimately controlling inhibition or excitation in the CNS. Although extensive studies have been performed to understand the pharmacological potential of inhibitory post-synaptic receptors, small molecules targeting scaffolding proteins such as gephyrin were unknown until recently. Recently, the anti-malarial drug artemisinin and its semi-synthetic derivatives, collectively referred to as artemisinins, were discovered to target GABA_A_R signaling via an interaction with gephyrin in pancreatic cells. While one study concluded that this interaction mediates the trans-differentiation of glucagon-producing Tα cells into insulin-secreting Tβ cells, thus ascribing an anti-diabetic nature to these compounds (Li et al., 2017), a subsequent study (van der Meulen et al., 2018) failed to reproduce the trans-differentiation of these pancreas-derived cells and hence concluded that artemisinins mediate just the dedifferentiation of pancreatic Tα cells. In addition to their anti-parasitic activity, artemisinins have additionally been implicated in regulating the activity of multiple cellular pathways, including the modulation of a variety of cancers (Crespo-Ortiz and Wei, 2012; Tu, 2016). Despite the widespread applications of these compounds as drugs and effectors of cellular pathways, the molecular basis of their regulatory properties has so far remained elusive. Hence, future research should also be directed toward the elucidation of the mechanism of action of these anti-malarials on gephyrin. If these molecules indeed bind directly to gephyrin and also regulate neurotransmission, they could serve as potent gephyrin-specific modulators with therapeutic benefits against severe neurological disorders (Agarwal et al., 2008; Fang et al., 2011; Dejanovic et al., 2014a, 2015).

## Author Contributions

All authors listed have made a substantial, direct and intellectual contribution to the work, and approved it for publication.

## Conflict of Interest Statement

The authors declare that the research was conducted in the absence of any commercial or financial relationships that could be construed as a potential conflict of interest.
